# Evaluation of Hardness and Wear of Conventional and Transparent Zirconia Ceramics, Feldspathic Ceramic, Glaze, and Enamel

**DOI:** 10.3390/ma17143518

**Published:** 2024-07-16

**Authors:** Beata Dorota Dejak, Cezary Langot, Michal Krasowski, Marek Klich

**Affiliations:** 1 Department of Prosthetic Dentistry, Medical University of Lodz, 92-213 Lodz, Poland; cezarylangot@gmail.com; 2 University Laboratory of Materials Research, Medical University of Lodz, 92-213 Lodz, Poland; michal.krasowski@umed.lodz.pl; 3 Institute of Materials Science and Engineering, Technical University, 90-924 Lodz, Poland; marek.klich@p.lodz.pl

**Keywords:** tribological properties, ceramic wear, enamel wear, coefficient of friction, 3Y-TZP, 5Y-TZP

## Abstract

The aim of the study was to compare the hardness, coefficient of friction, and wear experienced by four different ceramic samples: 3Y-TZP zirconium oxide ceramics—Zi—Ceramill Zi (Amman Girrbach), 5Y-PSZ transparent zirconium oxide ceramics—Zol—Ceramill Zolid (Amman Girrbach), Sak—feldspathic ceramics—Sakura Interaction (Elephant), and Glaze (Amman Girrbach). The Vickers hardness of the samples was measured. Friction tests ball-on-disc were performed between the discs of four ceramics and a zirconia ceramic ball, then a premolar tooth as a counter-sample. The mass loss and the friction coefficients of the ceramic samples were determined. The tooth counter-samples were 3D scanned, and enamel attrition depths and mass were measured. The following hardness values (HV1) were obtained: 1454 ± 46 HV1 for Zi, 1439 ± 62 HV1 for Zol, 491 ± 16 HV1 for Sak, 593 ± 16 HV1 for Glaze, and 372 ± 41 HV1 for enamel. The mass losses of the teeth in contact with ceramics were 0.1 mg for Zi, 0.1 mg for Zol, 5.5 mg for Sak, and 4 mg for Glaze. Conventional and transparent zirconium oxide ceramics are four times harder than enamel and three times harder than veneering ceramics. Zirconia ceramics exhibit lower wear and a more homogenous, smoother surface than the other ceramics. Tooth tissues are subject to greater attrition in contact with veneering ceramics than with polished zirconium oxide ceramics.

## 1. Introduction

Modern dentistry employs various ceramics, such as feldspar, leucite, lithium disilicate and zirconium oxide to make fixed dentures. Due to their strength, the first three are used for inlays, onlays, and single crowns. Zirconium oxide ceramics are used to make veneered or monolith crowns and bridges.

Zirconia ceramic is a polymorphic material occurring in three allotropic varieties: monoclinic (m), tetragonal (t) and cubic (c) (depending on temperature). 3Y-TZP (3 mol% yttria stabilized tetragonal zirconia polycrystal) consists of tetragonal crystals stabilized at room temperature by adding yttrium oxide [1]. ZrO_2_ crystals form grains measuring around 0.5–1 µm. This ceramic has the highest flexural strength (900–1200 MPa), is very stiff (with Young’s modulus of 210 GPa), and very hard [2]. Zirconia ceramic exhibits good mechanical properties and it can replace metal in prosthetic restorations. Studies indicate that the veneered three-unit FPDs (fixed partial dentures) of 3Y-TZP are able to carry twice as much load (up to 2000 N) as FPDs of another ceramics (below 1000 N) [3]. Tetragonal crystals of 3Y-TZP are birefringent, which makes this material opaque. Therefore, they are used as the cores of restorations, which are then veneered with transparent feldspar ceramics [4]. Unfortunately, in 6–15% of cases, the veneering ceramic chips move away from the core over a period of three to five years; this complication was observed in 4% of cases over a period of 10 years in metal–ceramic crowns [5].

In recent years, transparent zirconium oxide ceramics have been designed for use in full-contour crowns and bridges. The material 4-6Y-PSZ (4–6 mol% yttria partially stabilized zirconia) contains an increased amount, 9.3 wt%–5 mol%, of yttrium oxide [6]. To achieve greater transparency, it should be sintered at a temperature of 1300–1600 °C and cooled rapidly. As a result of this process, a larger amount of the cubic phase remains in the structure of the ceramic [7]. Cubic crystals of 5Y-PSZ create big grains, have low porosity, and have an isotropic refractive index. This ceramic has similar transparency and color to tooth tissue; hence, it does not require additional ceramic coating. Consequently, this allows for less tooth preparation and simpler laboratory procedures, and the production of highly durable and more aesthetic restorations [8]. Unfortunately, 5Y-PSZ has lower strength because the strengthening transformation phenomenon is limited due to the small amount of tetragonal phase [9].

Restorative materials should have wear resistance similar to tooth enamel. Most studies indicate that the wear resistance of zirconium oxide ceramics is very low [9]. Current research does not allow us to clearly state how transparent zirconia ceramic reacts in contact with other restorations and with the enamel of antagonist teeth [10,11,12]. Clinically, the attrition process is influenced by many parameters, including the type of consumed food, occlusal habits and parafunctions, hardness and roughness of the material covering the antagonist teeth, and the quality of saliva [13,14]. There is therefore a need to confirm whether 3Y-TPZ veneered with feldspar ceramics, or monolithic polished or glazed 5Y-PSZ ceramics is more beneficial for opposing teeth.

The aim of this study was therefore to compare the hardness, surface roughness, the coefficient of friction, and abrasive wear demonstrated by conventional and transparent zirconia ceramics, feldspathic ceramics, glaze, and enamel.

## 2. Materials and Methods

Samples were taken of a 3Y-TPZ zirconium oxide ceramic (Zi group), i.e., Ceramill Zi (Amman Girrbach, Koblach, Austria); a 5Y-TZP transparent zirconium oxide ceramic (Zol group), i.e., Ceramill Zolid (Amman Girrbach, Koblach, Austria); a feldspathic ceramic (Sak group), i.e., Sakura Interaction (Elephant Dental, Dentsply, The Nederlands); and a glaze (Glaze group), i.e., Glaze (Amman Girrbach, Koblach, Austria) (Table 1). These materials are often used in the clinic. Four discs, 25 mm in diameter and 4.5 mm thick, were designed in Ceramill Mind engine build 5049 CAD software, milled in a Ceramill Motion 2 from Zi and Zol ceramics, then sintered in a Ceramill-Therm 3 (Amman Girrbach, Koblach, Austria) furnace. Sak and Glaze ceramics were used for veneer, so they were sintered onto two of the discs. A ceramic ZrO_2_ ball and premolar teeth were used as counter samples.

The surface hardness of the four samples was measured using the Vickers method with a ZHµ M semi-automatic hardness tester (Zwick/Roell, Ulm, Germany), calibrated to PN-EN ISO 6507:1-3 standard [15]. Briefly, a Vickers-type diamond indenter, shaped like a pyramid with a square base and an angle α = 136°, was pressed into the surface of the tested sample. Five measurements were taken for each material at a load of approximately 10 N and within a period of 10 s.

**Figure 1:**
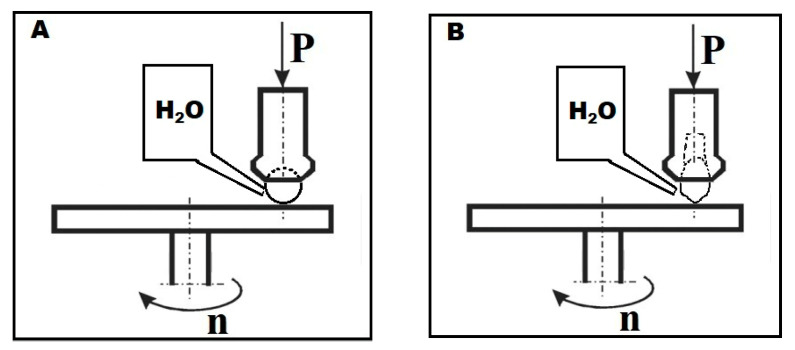
Diagram of friction systems for (**A**) ceramic ball and (**B**) tooth.

Prior to tribological testing, the surfaces of all samples were made uniform using a Struers polishing machine with an MD-Allegro diamond disc. Two friction-pair systems (ZrO_2_ ceramic and teeth) were created to test the attritive wear resistance of the surfaces of the four ceramics. The tribological properties were determined using a T-11 Tester (Instytut Technologii Eksploatacji Państwowy Instytut Badawczy, Radom, Poland), based on the ASTM G99-17 standard [16], operating as ball-on-disc and pin-on-disc systems in an aqueous environment (demineralized water, 25 mL) at 36 °C. The diagram of the friction systems is shown in Figure 1.

In the first stage of the study, the behavior of samples was determined in the ball-on-disc friction test, using a 6.35 mm diameter ball (Redhill balls, Prague, Czech Republic), made of ZrO_2_ (99.9%) as a counter-sample material (Figure 1A). The ball moved along a circle with a radius of 7 mm, covering a distance of 1000 m. A constant 10 N load was applied throughout the measurement. The results were used to determine the coefficient of friction and mass loss of the samples.

Following this, the surfaces of the attrited discs were analyzed with a SJ-410 surface tester (Mitutoyo, Sakado, Japan). Six primary profile measurements were taken perpendicular to the friction path. The results were used to determine the depth and width of the attrition. The surface condition of the ceramic samples was also assessed using a BX51 optical microscope (Olimpus, Tokyo, Japan) and Eclipse MA200 metallographic microscope (Nikon, Tokyo, Japan).

Surface geometry was also determined prior to and after tribological testing with a model SJ-410 profilometer (Mitutoyo, Sakado, Japan). Roughness parameters were determined in compliance with the ISO-4287:1997 standard [17]. The measuring section Lt was 4 mm long and consisted of five elementary sections Lc = 0.8 mm. The needle travel speed was v = 0.2 mm/s. Various surface roughness parameters were determined, and Ra and Rz were subject to analyses.

The second stage evaluated the degree of attrition of the enamel of premolar teeth while in contact with the four ceramics. The test was carried out using a T-11 Tester (Instytut Technologii Eksploatacji Państwowy Instytut Badawczy, Radom, Poland) using a pin-on-disc system, in which the tooth was positioned at an angle of 45° to the sample surface (Figure 1B). Due to the relatively low hardness of the enamel, a constant load of 5 N was applied to the friction node over a friction distance of 500 m and a speed of 0.1 m/s. In order to reduce the influence of surface preparation on the results, the tests were carried out using samples from the previous test, keeping a two-millimeter gap between the friction paths of the ball and the tooth. The mass loss was calculated for the counter-sample from the tooth. The tooth samples were scanned in a Cerramill Map 300 scanner (Amman Girrbach, Koblach, Austria) before and after testing. The depths of enamel attrition in contact with different ceramics were assessed by superposing 3D images of the teeth taken before and after friction in .stl files and analyzing the 3D measurement data with GOM Inspect V 7.5 SR2 software (Capture 3D a Zeiss Company, Santa Ana, CA, USA). The volume of the attrited teeth was also determined to calculate the wear coefficients of the tested materials.

## 3. Results

**Figure 2:**
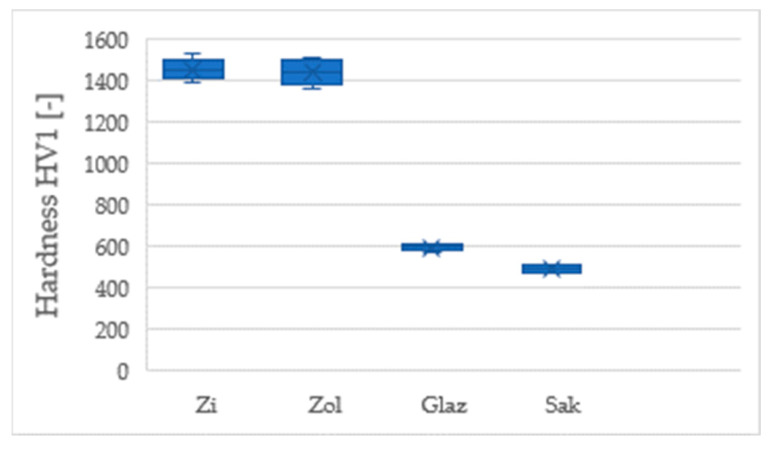
Comparison of the Vickers hardness of tested ceramic materials.

The Vickers test found the conventional and transparent zirconium oxide to have similar hardness values, viz. 1454 ± 46 HV1 and 1439 ± 62 HV1, respectively. In contrast, the Sakura Interaction ceramic (Elephant) was rated at 491 ± 16 HV1 and the Glaze–Glaze group (Amman Girrbach) at 593 ± 16 HV1. The hardness of the enamel was 372 ± 41 HV1. These data are presented in Figure 2.

**Figure 3:**
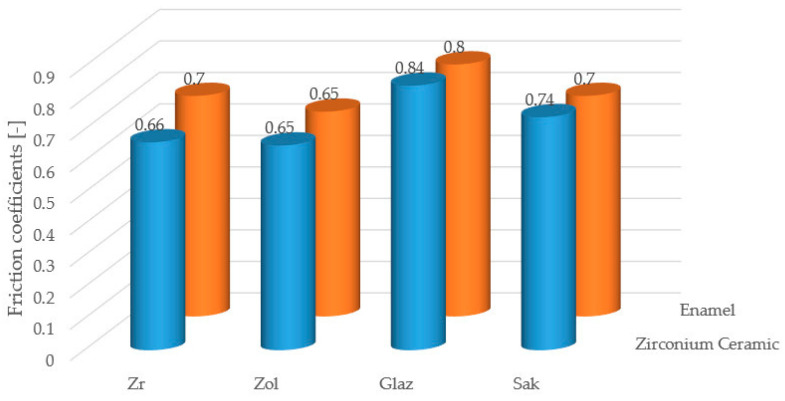
Comparison of the friction coefficients of the tested ceramics in contact with a counter-sample in the form of zirconium ball and enamel.

The following friction coefficients were recorded against the opposing zirconia ball: Zi—0.66, Zol—0.65, Sak—0.74, Glaze—0.84. The following values were obtained against the enamel: Zi—0.7, Zol—0.65, Sak—0.7, Glaze—0.8 (Figure 3). It should be noted that the friction coefficient Zol was the lowest of the ceramics tested.

**Figure 4:**
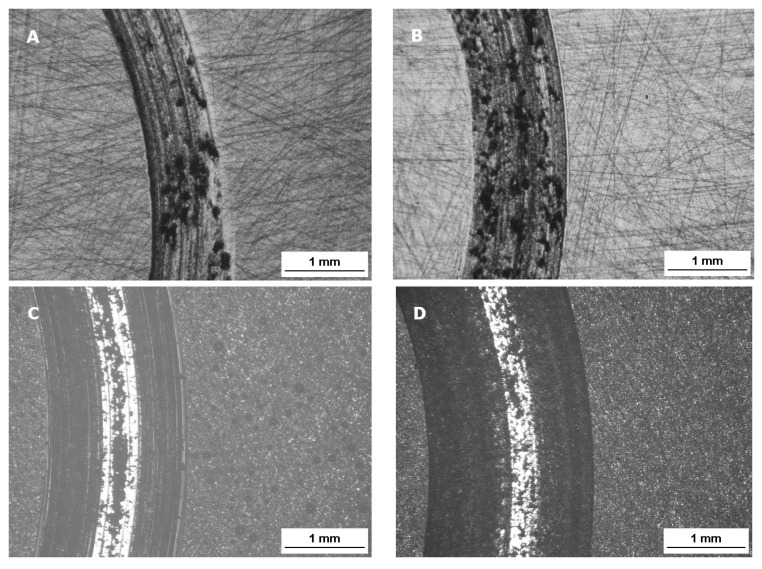
The surface layer of the ceramic samples after friction testing by contact with a zirconium ball counter sample: (**A**) Zr—zirconium oxide—Ceramill Zi (Amman Girrbach), (**B**) Zol—zirconium oxide Ceramill Zolid (Amman Girrbach), (**C**) Glaze (Amman Girrbach), (**D**) Sak–Sakura Interaction feldspar ceramics (Elephant). Images taken with a BX51 optical microscope (Olympus) with 5× magnification.

Attrition of the ceramic surface after friction: microscope images (Figure 4). The Sak and Glaze friction images show abraded depositors of the materials.

**Figure 5:**
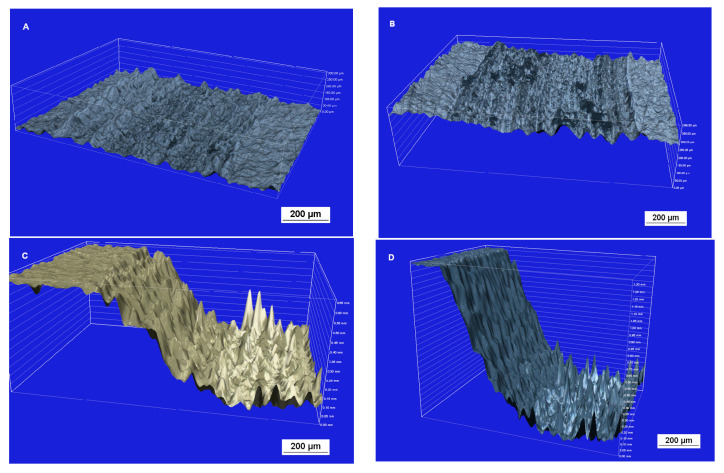
Surface layer profiles of samples after friction testing against a zirconium ball counter-sample: (**A**) Zi—zirconium oxide—Ceramill Zi (Amman Girrbach), (**B**) Zol—zirconium oxide Ceramill Zolid (Amman Girrbach), (**C**) Glaze (Amman Girrbach), (**D**) Sak—Sakura Interaction feldspar ceramics (Elephant). Images taken with a Nikon Eclipse MA200 (Nikon) metallographic microscope with a confocal attachment.

After the attrition resistance test, the attrited surface of the ceramics was assessed using an inverted optical metallographic microscope (Figure 5). Feldspar ceramics and glaze had the most worn and uneven surfaces due to friction. In contrast, both zirconium oxide ceramics had similar, smooth surfaces.

Among the tested zirconium oxide materials, Zi and Zol demonstrated similar attrition width (1.11 ± 0.14 mm and 1.12 ± 0.18 mm, respectively). These values were significantly lower than those noted for Glaze (1.91 ± 0.22 mm) and Sak (2.06 ± 0.19 mm). However, considerable differences in depth of attrition were observed: 6.2 ± 0.4 mm for Zi, 7.8 ± 1.7 mm for Zol, 90 ± 8.4 mm for Glaze, and 126 ± 23.3 mm for Sak. The losses in mass were found to be 1.4 ± 0.1 mg for Zi, 2.5 ± 0.1 mg for Zol, 13.9 ± 0.1 mg for Glaze, and 23.4 ± 0.1 mg for Sak. The losses in mass demonstrated by the ceramics during the test are compared in Table 2. The mass loss of Zi ceramics was almost twice as small as that of Zol. However, Sak attrition was 10 times greater.

**Figure 6:**
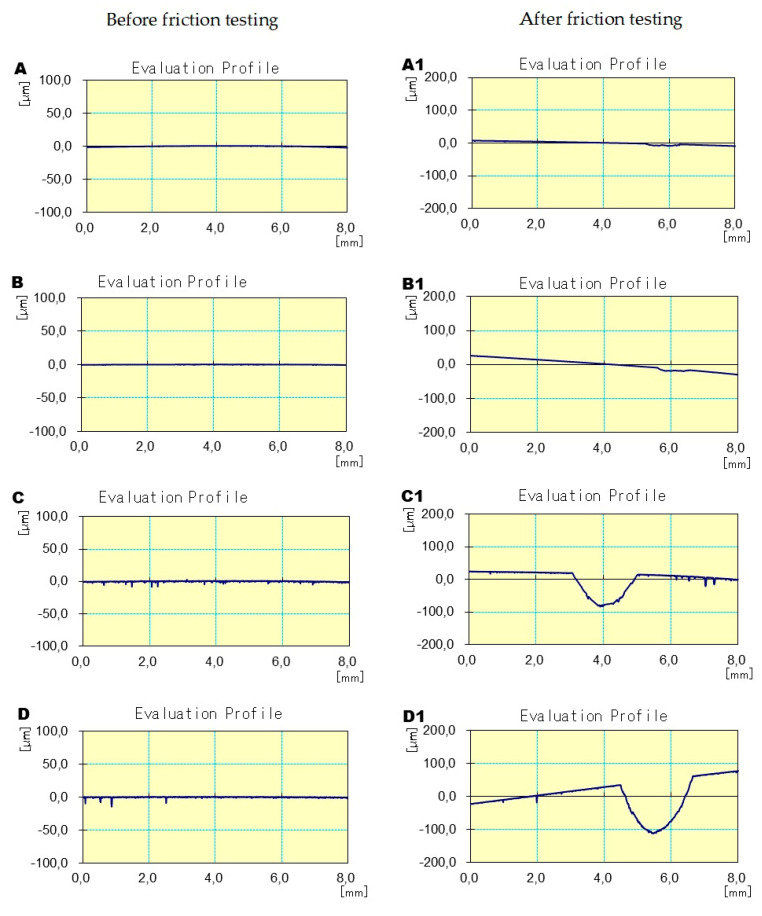
Example primary profiles showing the depth and width of abrasion before and after friction testing in contact with the zirconium ball counter-sample: (**A** before friction, **A1** after test) Zi—zirconium oxide—Ceramill Zi (Amman Girrbach), (**B** before friction, **B1** after test) Zol—zirconium oxide Ceramill Zolid (Amman Girrbach), (**C** before friction, **C1** after test) Glaze (Amman Girrbach), (**D** before friction, **D1** after test) Sak—Sakura Interaction feldspar ceramics (Elephant).

The next stage evaluated the surface roughness profiles of the ceramics in the attrition area. Increases in the values of the tested parameters were observed for all materials. However, this increase was much greater for the feldspathic ceramics and the glazes than in cases of Zi and Zolid materials (Table 3) (Figure 6).

**Figure 7:**
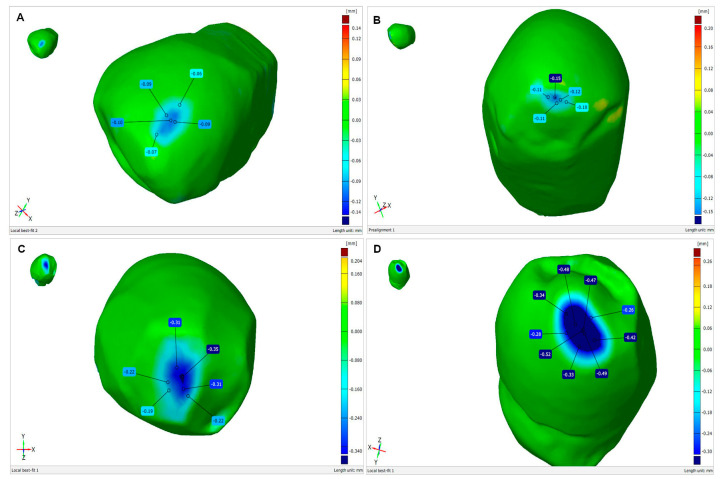
Abrasive wear of premolars during friction testing in contact with ceramics: (**A**) Zi—zirconium oxide—Ceramill Zi (Amman Girrbach), (**B**) Zol—zirconium oxide Ceramill Zolid (Amman Girrbach), (**C**) Glaze (Amman Girrbach), (**D**) Sak—Sakura Interaction feldspar ceramics (Elephant). Images obtained by GOM Inspect V 7.5 SR2 software.

The attrition experienced by the tooth samples is illustrated by superposed 3D scans taken before and after friction testing in GOM Inspect V 7.5 SR2 software (Figure 7).

The depth of the enamel loss was 0.09 mm for Zi ceramic, 0.15 mm for Zol ceramic, 0.31 mm for glaze and 0.52 mm for Sak ceramic. The reductions in mass demonstrated by the tooth in contact with the ceramics were 0.1 ± 0.1 mg for Zol, 0.1 ± 0.1 mg for Zi, 5.5 ± 0.1 mg for Sak, 4 ± 0.1 mg and Glaze (Table 4).

## 4. Discussion

Our data from the Vickers hardness test indicate that conventional and transparent zirconium oxide ceramics have similar hardness. These ceramics are the hardest among the tested materials. Unfortunately, both materials are three times harder than veneering ceramics and about four times harder than enamel, which increases the wear placed on the tooth. Previous studies have found the zirconia ceramics' hardness to be about 1350 HV [18] and 1378.7 HV [19].

The Zolid zirconium oxide ceramics demonstrated the lowest friction coefficient (0.65) against ceramic or teeth counter samples. This coefficient differed slightly from that for the conventional Zi ceramic (0.66). However, it was significantly lower than conventional feldspar and glaze ceramics (Sakura Interaction—0.74, Glaze—0.84). The surface of zirconium oxide ceramics glides easily over other materials as well as enamel.

The surface of the polished Zol zirconium oxide ceramics was not very developed and was smooth (Ra 0.24 µm); this was most likely due to its homogeneous structure, which consists of small 0.1–1 µm grains that strongly adhere to each other after sintering. In contrast, surfaces of conventional ceramics were 20 times rougher after wear (Ra 5224 µm). The surface of feldspar ceramics was characterized by large irregularities and chips. Feldspathic or leucite ceramics contain crystalline grains of silicates and leucite (about 10 µm) embedded in amorphous silica. The glass matrix is abraded in the mastication process, thus exposing large crystal grains, which grind down the antagonist teeth. Glaze forms a thin layer of approximately 0.1 mm, which is abraded unevenly during mastication; this roughens the surface of the restoration [20]. A SEM study by Park et al. [21] also observed that, after a friction test, the surface of feldspathic ceramics and glaze contained blisters and pores, whereas that of zirconia oxide appeared to be smooth and homogenous.

The Zi conventional and Zol transparent zirconia ceramics showed similar minimal mass losses in contact with a ZrO_2_ ceramic counter sample; these values were 9–16 times lower than those of glazes and leucite ceramics. Zurek et al. stated that zirconia demonstrated greater resistance to wear and lower abrasiveness compared to other ceramics in a simulated masticatory environment [22]. Both Lopez et al. and Amera et al. [23,24] report that zirconia ceramics exhibited the lowest attrition among the tested substances.

Our findings indicate that the enamel in contact with zirconia ceramic, especially polished Zolid, undergoes low attrition. In contrast, the feldspathic ceramics and glaze demonstrated many times greater wear of the enamel compared to zirconium oxide ceramics. This is confirmed by other studies. Park et al. [21] report lower attrition of enamel in contact with polished Y-TZP zirconium oxide ceramics (1.11 mm^3^) compared to glaze (3.07 mm³). Zandpars et al. [25] found Lava Plus Zirconia ceramics to demonstrate lower enamel attrition (27.5 mm^3^) compared to other ceramics: and feldspathic Noritake Super Porcelain EX-3 system (34.75 mm^3^). In a study based on simulated mastication cycles, Chong et al. [26] found that crowns made of zirconium oxide ceramics adjusted to occlusion with diamond drills attrited tooth enamel in a similar way to natural teeth. Polished zirconium oxide is the least attritive to tooth enamel compared to zirconium ceramic veneered with glaze or porcelain [27,28], and demonstrates the least abrasion to antagonist teeth [29,30,31].

A two-year clinical observational study of tooth wear under natural conditions by Pintado et al. [32] found the mean enamel wear volume to be 0.04 mm^3^ and depth to be 10 µm. Mundhe et al. [33] clinically studied tooth attrition in contact with opposing natural teeth, or metal–ceramic or zirconia crowns, for one year. Zirconia crowns appeared to cause lower attrition of molar antagonist teeth (127.0 µm) than metal–ceramic crowns (179.7 µm), but significantly higher attrition of natural teeth (35.1 µm). A clinical study by Strober et al. [34] confirms that over a period of six months, zirconium oxide crowns demonstrated greater attrition of tooth enamel (33 µm) than natural antagonist teeth (10 µm). Tang et al. [35] found that a monolithic zirconia crown can cause significant wear of the antagonist teeth via occlusal or early contact; this wear increases over time. The wearing mechanism is mainly abrasive and based on fatigue wear.

In dentistry, various tribological research methods are used: a cylinder-cusp pair of materials, placed in a chewing simulator, undergoes cycles 10,000–300,000 under a load of 10–50 N [9,12]. Studies are also carried out using a reciprocating tribometer with a force of 20 N and stroke length of 2 mm [36]. In this study, tribological tests were carried out on a laboratory tester, based on the ASTM G99-17 standard [16]. Research conducted this way is repeatable and standardized. The experiment was performed once. This was due to the fact that the pin-on-disk method has not been used to test tooth–ceramic friction so far. These investigations should be treated as pilot studies. Future research should track the evolution of wear or mass loss over time.

Zirconium oxide ceramic, regardless of its modification, is a very hard and wear-resistant material. When making monolithic zirconium oxide crowns in antagonist dental arches, it is important to remember that their surface will suffer minimal adaptive attrition. Surface of 5Y-PSZ is smooth, the friction coefficients of 5Y-PSZ in contact with other materials and teeth is very low. Therefore, polished zirconium oxide surfaces contribute to less wear of antagonist teeth compared to other ceramics [37] and to veneered zirconium oxide ceramic restorations. Unfortunately, the enamel in contact with the ceramic experiences greater wear compared to contact with natural teeth.

## 5. Conclusions

Within the limitations of this study, the following conclusions were drawn:

1. Conventional and transparent zirconium oxide ceramics are very hard, being four times harder than enamel and three times harder than veneering ceramics.
2. The friction coefficient of zirconium oxide ceramics, especially polished 5Y-PSZ, is lower than that of leucite ceramics and glazes.
3. The surface of zirconium oxide ceramics is smooth and homogeneous after attrition compared to other ceramics.
4. Conventional and transparent zirconia ceramics exhibit minimal wear compared to that observed for veneering ceramics.
5. The enamel suffers greater attrition in contact with veneering and glazing ceramics than with polished zirconium oxide ceramics.
6. The pin-on-disc method can be used to test the wear of dental materials.

## Figures and Tables

**Table 1:** Materials used in study.

Material	Manufacturer	Technical Data
Ceramill Zi	Amman Girrbach, Koblach, Austria	3 mol% yttria-stabilized tetragonal zirconia polycrystal
Ceramill Zolid	Amman Girrbach, Koblach, Austria	5 mol% yttria partially stabilized zirconia
Sakura Interaction	Elephant Dental Dentsply, The Nederlands	Feldspathic ceramic
Glaze	Amman Girrbach, Koblach, Austria)	Glass ceramic

**Table 2:** The width and depth of the attrition and mass loss from the surface after the attrition test.

Ceramic Materials	Wear Width (mm)	Wear Depth (mm)	Mass Loss (mg)
Zr	1.11 ± 0.14	6.2 ± 0.4	1.4 ± 0.1
Zol	1.12 ± 0.18	7.8 ± 1.7	2.5 ± 0.1
Glaze	1.91 ± 0.22	90.0 ± 8.4	13.9 ± 0.1
Sak	2.06 ± 0.19	126.0 ± 23.3	23.4 ± 0.1

**Table 3:** Surface roughness of ceramic samples before and after tribological testing.

Ceramic Materials	After Tribological Testing			After Tribological Testing		
	Ra µm	Rq µm	Rz µm	Ra µm	Rq µm	Rz µm
Zi	0.038	0.049	0.390	0.337	0.104	2.183
Zol	0.029	0.038	0.257	0.240	0.310	1.613
Glaze	1.093	1.317	5.188	3.777	5.316	25.224
Sak	0.533	0.596	1.440	5.224	7.169	33.494

**Table 4:** Abrasive wear of enamel after pin-on-disc testing in contact with ceramics.

Ceramic Materials	Wear Deep (mm)	Mass Loss (mg)
Zi	0.09 ± 0.01	0.1 ± 0.1
Zol	0.15 ± 0.01	0.1 ± 0.1
Glaze	0.35 ± 0.01	4 ± 0.1
Sak	0.12 ± 0.01	5.5 ± 0.1

## Data Availability

The original contributions presented in the study are included in the article, further inquiries can be directed to the corresponding author.
